# Adverse drug reactions to atezolizumab in combination with bevacizumab in hepatocellular carcinoma patients: an analysis of the food and drug administration adverse event reporting system database

**DOI:** 10.3389/fphar.2025.1448095

**Published:** 2025-02-21

**Authors:** Wanming He, Lihua Tong, Yanling Yuan, Xia Yang, Wen Yang, Xingxi Pan

**Affiliations:** Department of Oncology, The Sixth Affiliated Hospital, School of Medicine, South China University of Technology, Foshan, China

**Keywords:** anti-vascular endothelial growth factor, immune checkpoint inhibitor, programmed cell death protein 1, adverse events, hepatocellular carcinoma

## Abstract

**Purpose:**

The objective of the study is to systematically identify and evaluate the adverse drug reactions (ADRs) associated with the combination therapy of systematically and bevacizumab in patients with unresectable hepatocellular carcinoma (HCC).

**Patients and methods:**

Data were extracted from the Food and Drug Administration (FDA) Adverse Event Reporting System FDA Adverse Event Reporting System database. Disproportionality analysis was conducted using the reporting odds ratio (ROR), proportional reporting ratio (PRR) and Bayesian confidence propagation neural network (BCPNN) of information components (IC). Time-to-onset (TTO) profiles were analyzed using the Weibull shape parameter (WSP) test, while cumulative incidences were assessed using the Kaplan‒Meier method. Valuable preferred term (PT) signals were identified for designated medical event (DME) screening, comparing these signals with system organ class (SOC) analysis.

**Results:**

A total of 2,831 adverse events (AEs) reports were identified in the FAERS database, of which 124 positive AEs were detected across multiple SOCs. The median TTO of AEs was 43 days, with the highest proportion occurring within 0–30 days of TTO (n = 450, 41.17%). The WSP test indicated that patients with abnormal hepatic function and hepatic failure exhibited early failure-type profiles. Ten PT signals consistent with those on the DME list were identified, involving six SOCs.

**Conclusion:**

Our study provides valuable pharmacological insights for early clinical intervention in managing ADRs and offers significant clinical benefits for HCC patients undergoing combination therapy with atezolizumab and bevacizumab.

## Introduction

Hepatocellular carcinoma (HCC) is a prevalent malignancy and the third leading cause of cancer-related mortality worldwide (Balogh et al., 2016; Llovet et al., 2021; Sung et al., 2021). Although patients with early-stage disease can be effectively managed by surgical and locoregional treatments, approximately 70%–80% of patients are diagnosed at an advanced stage (Llovet et al., 2022), necessitating systemic therapies as the primary treatment modality.

Atezolizumab, an immune checkpoint inhibitor (ICI), targets programmed death 1 ligand 1 (PD-L1) to block its interaction with its receptors programmed cell death protein 1 (PD-1) and B7-1, which relieves the suppression of T cells and tumor immune escape (Herbst et al., 2014). Bevacizumab, a monoclonal antibody, inhibits angiogenesis and tumor growth by targeting anti-vascular endothelial growth factor (VEGF) (Boige et al., 2012; Ferrara et al., 2005; Finn et al., 2009; Siegel et al., 2008). The global, open-label, phase three IMbrave150 trial demonstrated that the combination of atezolizumab and bevacizumab significantly improved the median overall survival (OS) and progression-free survival (PFS) compared to sorafenib (Finn et al., 2020), a previously FDA-approved oral multikinase inhibitor for unresectable HCC (Lang, 2008; Llovet et al., 2008). Consequently, this combination therapy has been approved by the FDA over 70 countries as a first-line treatment for unresectable HCC in patients who have not received prior systemic therapy (Finn et al., 2020; Vogel et al., 2021; Yang et al., 2022).

With the increasing utilization of ICIs, immune-related adverse events (irAEs) have emerged as a significant challenge, limiting their clinical application and benefit. irAEs can affect multiple organ systems, including the skin, lungs, liver and endocrine tissues (Kennedy and Salama, 2020). Adverse drug reactions (ADRs) associated with bevacizumab include hypertension, asymptomatic proteinuria, bleeding, gastrointestinal perforation and thromboembolic events (Kazazi-Hyseni et al., 2010; Wichelmann et al., 2021; Ventura et al., 2023; Fukushima et al., 2023). However, the ADR profile of atezolizumab in combination with bevacizumab remains incompletely characterized. Given the complex biological interactions of this combined regimen, the potential for increased toxicity in HCC patients requires further investigation. Additionally, large-scale cohort studies evaluating this combination therapy are limited, which may not fully reflect real-world clinical outcomes.

Therefore, to investigate the ADRs of atezolizumab plus bevacizumab in HCC patients in depth, we conducted an analysis using the United States FDA Adverse Event Reporting System (FAERS) database (Rodriguez et al., 2001; van Puijenbroek et al., 2002), a large real-world database reporting hazardous drug events. Our aim is to guide early clinical intervention for managing ADRs and to provide clinical benefits for HCC patients undergoing this combination therapy.

## Materials and methods

### Data sources and procedures

The data for this study were obtained from the FAERS database, a spontaneous reporting system that collects information about adverse events (AEs) and medication errors reported to the FDA. It is free and publicly available online at https://open.fda.gov/data/faers/. No institutional ethics approval was required because this study utilized anonymized data from an open-access database.

### Data extraction

**FIGURE 1:**
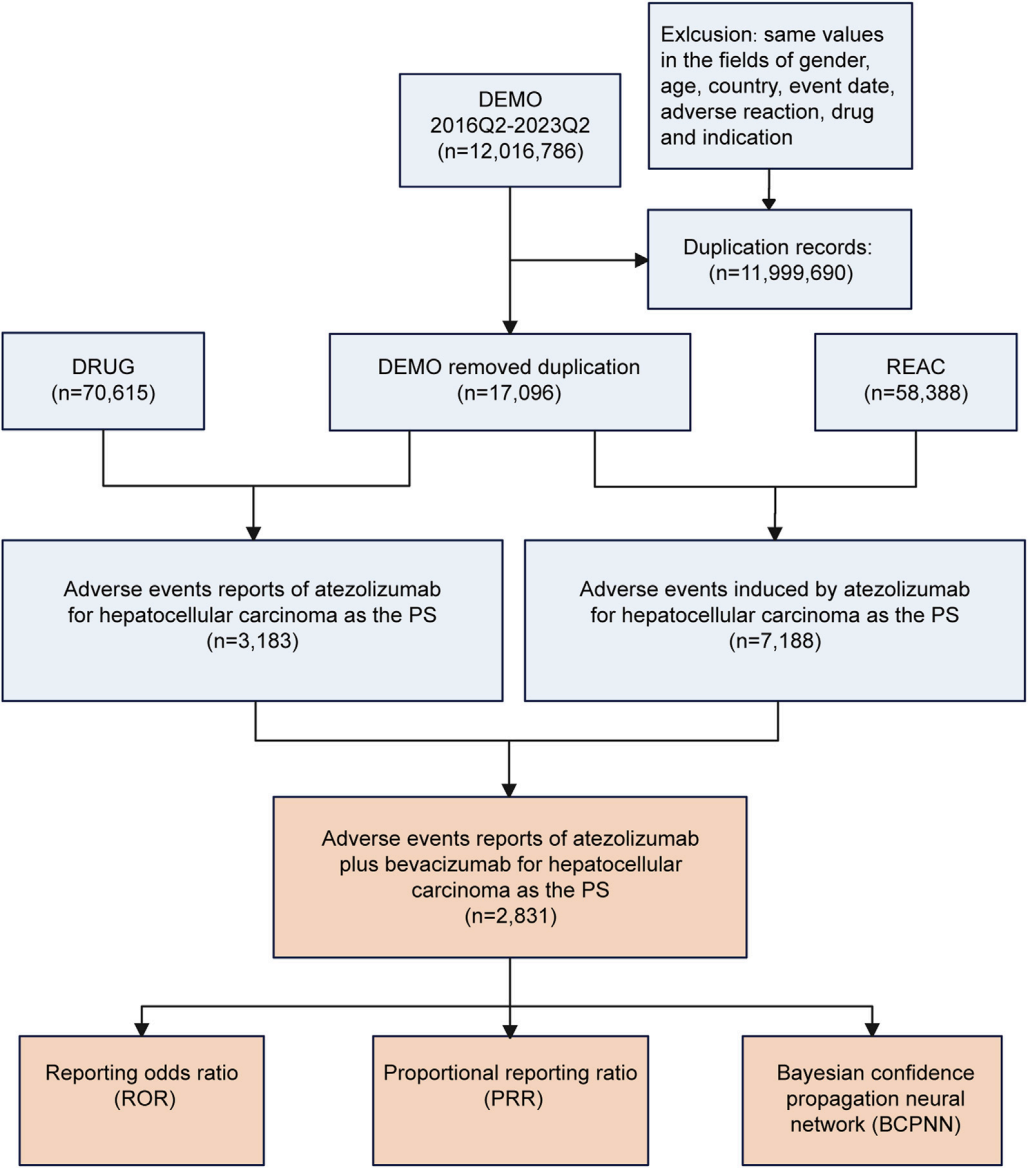
Flowchart for screening and data analysis of reports in the FAERS database. DEMO, demographic and administrative information; DRUG, drug information; REAC, preferred terminology for adverse events; PS, primary suspected drug.

To extract AE reports, we collected data from the FAERS database covering the period from the second quarter of 2016 to the second quarter of 2023 using the online tool OpenVigil 2.1 (http://openvigil.sourceforge.net/). Only drugs listed as “primary suspects” were included in the analysis because they were most likely related to AEs. AEs were defined as adverse reactions in patients treated with atezolizumab combined with bevacizumab. AE reports are standardized using preferred terms (PTs) from the Standardized MedDRA Queries (SMQ) of the Medical Dictionary for Regulatory Activities (MedDRA Version 26.1), grouped by system organ class (SOC). Designated medical events (DMEs) were selected according to the lists developed by the European Medicines Agency (EMA) (Liu et al., 2023). The multistep process of data extraction and analysis is shown in Figure 1.

### Statistical analysis

Three algorithms, reporting odds ratio (ROR), proportional reporting ratio (PRR) and Bayesian confidence propagation neural network (BCPNN) of information components (IC), were used based on disproportionality analysis and Bayesian analysis. The equations and criteria for the three algorithms are described in Supplementary Table S1.

The time-to-onset (TTO) analysis was conducted using the Weibull shape parameter (WSP) test (Abe et al., 2016; Tisdale et al., 1995). The shape of the Weibull distribution was described by two parameters: scale (α) and shape (β). For our study, only the parameter β was used. The shape parameter β of the Weibull distribution indicated that the hazard without a reference population. When parameter β was <1 and its 95% CI was <1, the hazard of ADR occurrence was deemed to have decreased over time (early failure-type profile); when parameter β was equal to or nearly 1 and its 95% CI included value 1, the hazard was regarded as constant occurrence over time (random failure-type profile); and when parameter β was >1 and its 95% CI excluded value 1, the hazard was estimated to increase over time (wear-out failure-type profile) (Kinoshita et al., 2020; Nakamura et al., 2015; Sauzet et al., 2013). Additionally, to further determine whether age and sex had effects on TTO, cumulative incidences were assessed using the Kaplan‒Meier method, with differences determined using the log-rank test. A statistically significant threshold was set at a p value of <0.05 for all analyses. All the analyses were performed in R (version 4.2.3; R Foundation, Vienna, Austria).

## Results

### Baseline characteristics of patients

A total of 12,016,786 AEs were recorded in the FAERS database from the second quarter of 2016 to the second quarter of 2023. After removing duplicates, a total of 17096 case reports of the primary suspected (PS) drug were included. Overall, 2,831 reports of atezolizumab and bevacizumab were included in the analysis. The process flowchart is shown in Figure 1.

The baseline characteristics of patients are summarized in Table 1. There was a greater proportion of male patients (72.6%) than female patients (13.8%), and sex information was missing for 13.6% of the patients. The largest proportion of patients were aged 65–85 years. These reports were collected mainly from Japan (63.9%), followed by the United States (3.6%) and China (3.2%). The majority of cases were reported by physicians (83.1%). The number of reports increased annually during the 2021–2023 period (31.5%, 38.9%, and 23.2%, respectively), indicating that combination therapy with atezolizumab and bevacizumab is widely used in the clinic. Hospitalization occurred in 31.4% of all patients, and death occurred in 17.5%.

**TABLE 1:** Clinical characteristics of HCC patients treated with atezolizumab in combination with bevacizumab.

Clinical characteristics	N	%
Total	2,831	
Sex		
Male	2054	72.6%
Female	391	13.8%
Missing	386	13.6%
Age		
18–64	466	16.5%
65–85	1042	36.8%
≥86	53	1.9%
Missing	1270	44.9%
Reporting country		
Japan	1809	63.9%
United States	101	3.6%
China	90	3.2%
India	83	2.9%
France	81	2.9%
Other countries	663	23.4%
Unknow	4	0.1%
Reporter type		
Physician	2,353	83.1%
Pharmacist	169	6.0%
Consumer	166	5.9%
Health-professional	143	5.1%
Reporting year		
2018	4	0.1%
2019	49	1.7%
2020	130	4.6%
2021	891	31.5%
2022	1101	38.9%
2023	656	23.2%
Outcome^a^		
Other serious	1569	46.1
Hospitalization	1070	31.4%
Death	595	17.5%
Life-Threatening	96	2.8%
Disability	27	0.8%
Missing	50	1.5%

^a^ Multiple responses were possible in each report.

### Disproportionality analysis

**FIGURE 2:**
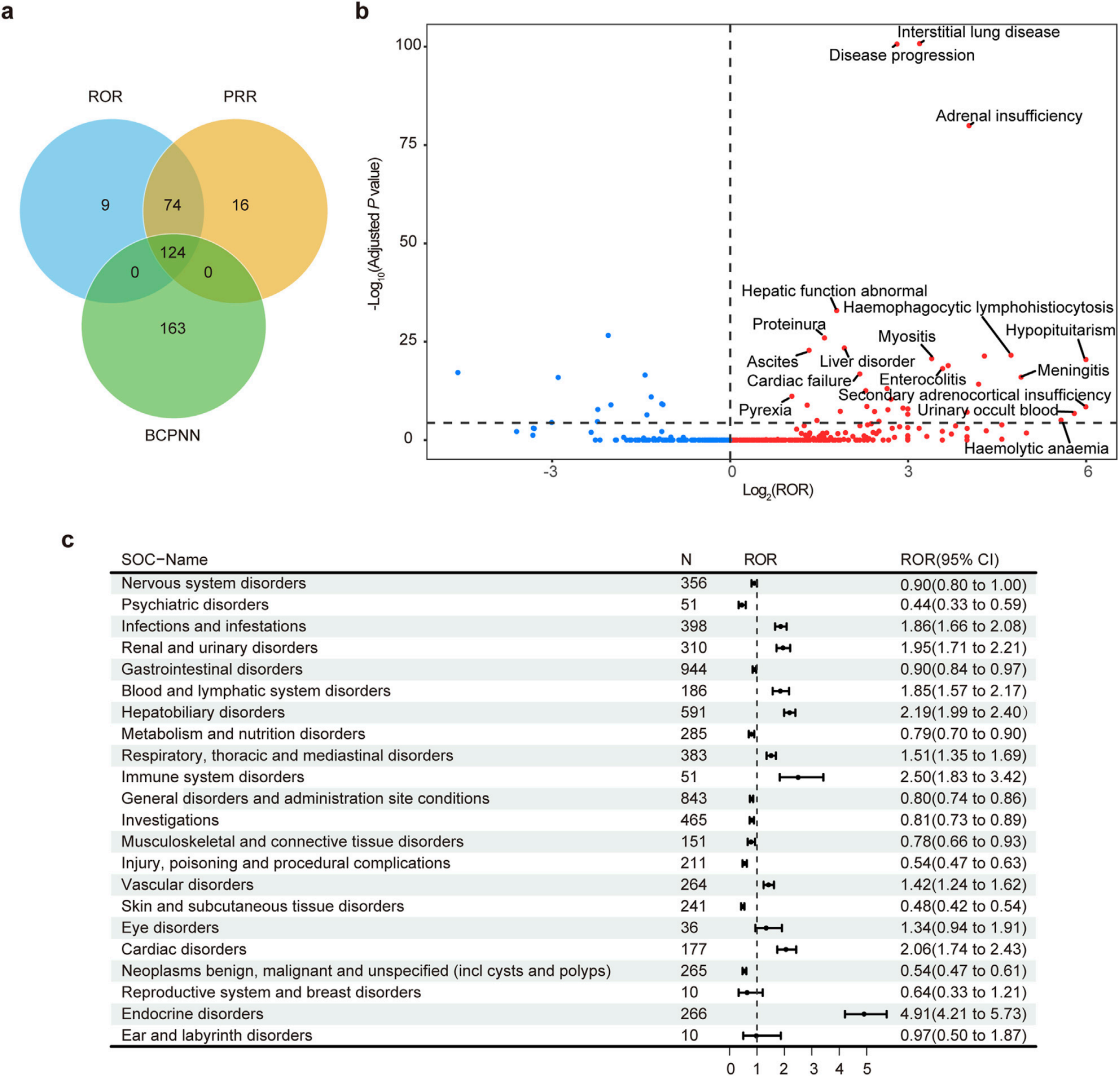
Adverse events (AEs) and system organ classes (SOCs) of atezolizumab in combination with bevacizumab. **(A)** Venn diagram showing the three algorithms used for disproportionality analysis. **(B)** The volcano plot shows the 1114 AEs. *P* values were adjusted with Bonferroni test. Adjusted *P* value = 4.89 × 10^−5^. **(C)** The forest plot shows the SOCs of AEs. ROR, reporting odds ratio; PRR, proportional reporting ratio; BCPNN, Bayesian confidence propagation neural network.

A total of 124 positive PTs was identified by three algorithms for disproportionality analysis and Bayesian analysis, namely, PRR, ROR and BCPNN of ICs (Figure 2A). A total of 54 PTs were identified as being related to atezolizumab, while three PTs were related to bevacizumab. Additionally, 27 PTs were found to be related to both drugs (Supplementary Table S2). The signal strength of all reported AEs was shown in Figure 2B. Hypopituitarism, secondary adrenocortical insufficiency, urinary occult blood, haemolytic anaemia and meningitis were the strongest five risk reports. The AEs were grouped according to SOC, and 22 organ systems were involved in atezolizumab- and bevacizumab-induced AEs (Figure 2C), of which ten significant SOCs were identified, including endocrine disorders, immune system disorders, hepatobiliary disorders, cardiac disorders, renal and urinary disorders, infections and infestations, blood and lymphatic system disorders, respiratory, thoracic and mediastinal disorders, vascular disorders and eye disorders. The most common SOC was hepatobiliary disorders (n = 591, 9.10%).

### TTO analysis

**FIGURE 3:**
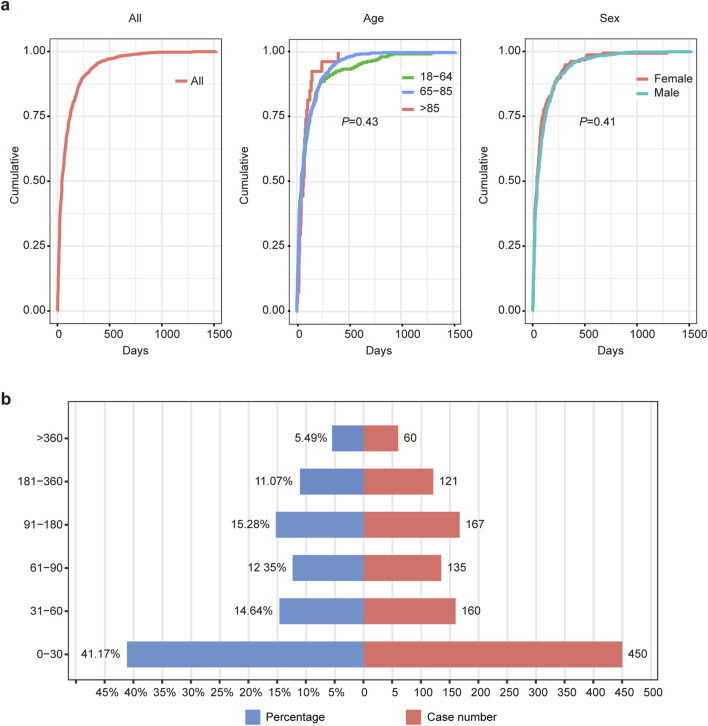
Time-to-onset (TTO) analysis of patients treated with atezolizumab plus bevacizumab. **(A)** Cumulative incidence of patients stratified by age and sex. **(B)** Number and proportion of patients stratified by time to onset.

After excluding patients with missing data, a total of 1093 patients were included, and the median TTO of AEs was 43 days (Table 2). To investigate the factors related to TTO, patients were stratified by age and sex. The median TTO in the 18–64, 65–85 and >85 years age groups were 43.5, 45 and 63, respectively (log-rank test, *P* = 0.4). Males had a median TTO of 45 days compared to 42 days for females (log-rank test, *P* = 0.4) (Table 2). However, there was no statistically significant difference in TTO among patients of different ages or sexes, indicating that age and sex are not factors influencing TTO due to AEs (Figure 3A). The number and proportion of patients stratified by TTO are shown in Figure 3B. The results indicated that the largest proportion (41.17%, n = 450) occurred in the 0–30 days TTO group.

**TABLE 2:** Time-to-onset analysis of patients stratified by age and sex.

		Age			Sex	
		18–64	65–85	>85	Male	Female
N	1093	274	570	27	879	156
Min TTO	1	1	1	1	1	1
Max TTO	1521	1307	1521	398	1521	1284
Median TTO	43	43.5	45	63	45	42
Logrank_test	-	*P* = 0.4			*P* = 0.4	

TTO, time to onset; N, number of patients with available TTO.

### WSP analysis

The WSP test is used for statistical analysis of time-to-onset data and can describe the non-constant rate of incidence of ADRs. Time-to-onset analysis with WSP has been used to evaluate hazard functions for detecting adverse events (Abe et al., 2016; Sauzet et al., 2013; Cornelius et al., 2012). To further assess severe AEs, WSP analysis of the top five PTs in terms of death outcomes is summarized in Table 3. The medians and quartile ranges (IQRs) for the onset day of disease progression, abnormal hepatic function, hepatic failure, interstitial lung disease and ascites were 42 (IQR: 20.5–92), 21 (7–66.25), 23 (10–70.5), 64.50 (26–117.25) and 50.50 (21–109) days, respectively. According to the WSP test for abnormal hepatic function and hepatic failure, the shape parameter β and the upper limit of the 95% CI were <1, suggesting an early failure-type profile, which indicated that the incidence of abnormal hepatic function and hepatic failure decreased over time. In the WSP test for disease progression, disease progression interstitial lung disease and ascites, the shape parameter β was nearly 1, and the 95% CI included the value 1, suggesting a random failure-type profile. This suggests the risks of disease progression, interstitial lung disease and ascites are almost constant.

**TABLE 3:** WSP analysis of the five PTs with the most deaths.

PT	Case (n)	TTO (days)		Weibull distribution				Failure type
				Shape parameter		Scale parameter		
		Median (IQR)	Min–max	α	95% CI	β	95% CI	
Disease progression	175	42 (20.50–92.00)	1–420	70.06	45.48–94.64	0.83	0.65–1.00	Random Failure
Hepatic function abnormal	57	21 (7.00–66.25)	1–370	46.14	31.55–60.73	0.80	0.65–0.94	Early failure
Hepatic failure	29	23 (10.00–70.5)	2–410	61.82	23.03–100.62	0.69	0.48–0.90	Early failure
Interstitial lung disease	25	64.50 (26.00–117.25)	1–607	94.34	67.81–120.87	0.95	0.77–1.13	Random Failure
Ascites	24	50.50 (21.00–109.00)	1–718	86.94	64.41–109.47	0.87	0.73–1.01	Random Failure

PT, preferred term; TTO, time to onset; n, number of patients with available TTO; IQR, interquartile range; CI: confidence interval.

## DME list screening

**FIGURE 4:**
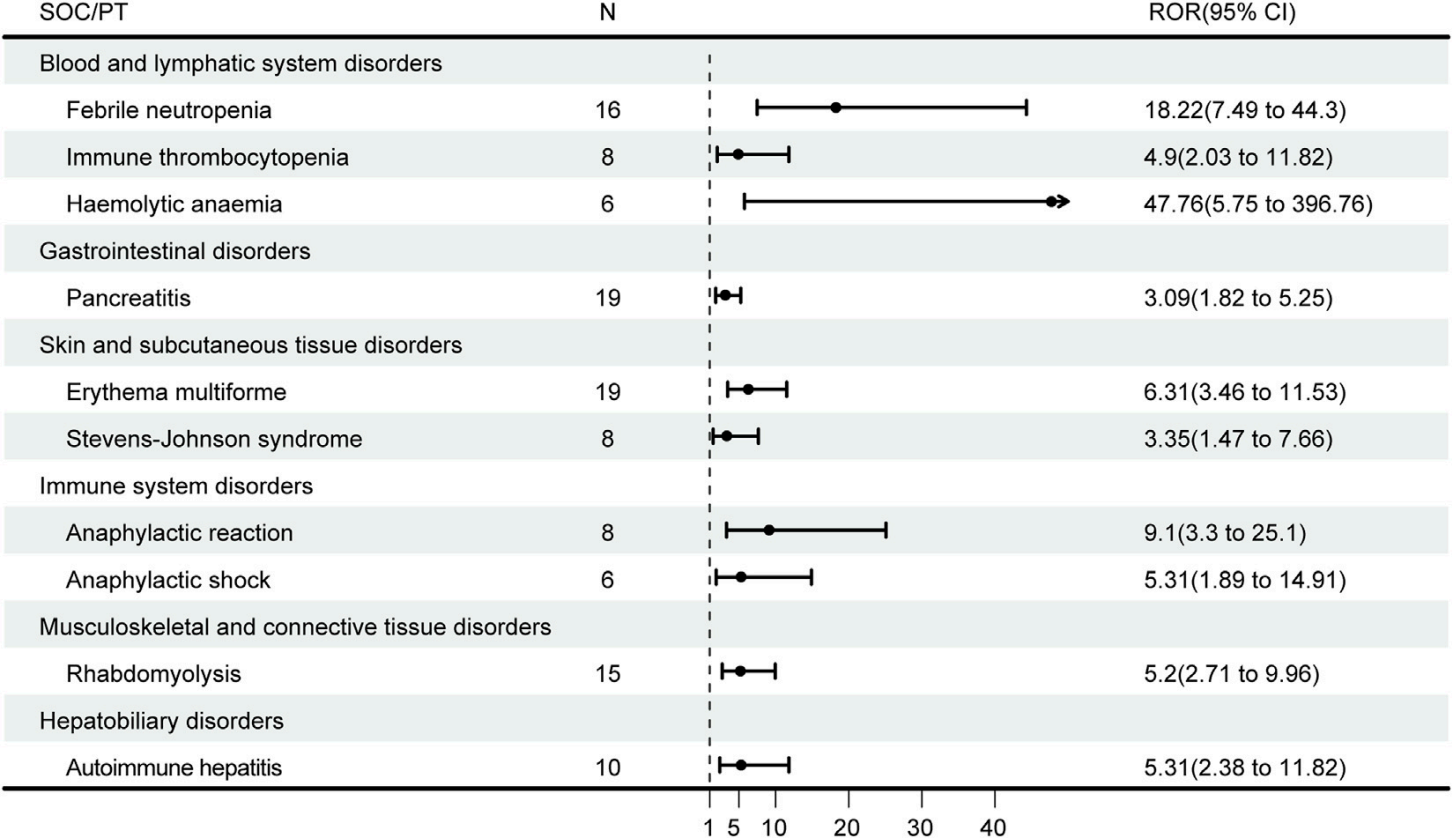
Detection of atezolizumab in combination with bevacizumab designated medical event (DME) signals. The forest plot showed that the DME signals and the SOCs corresponding to PT signals were consistent with those on the DME list. SOC, system organ class; PT, preferred term; ROR, reporting odds ratio; CI*:* confidence interval.

The DME is a list published by the EMA to identify suspected ADRs that deserve special attention (Liu et al., 2023). The valuable positive signals extracted for DME screening and the SOCs corresponding to PT signals consistent with those on the DME list are shown in Figure 4. There are ten signals consistent with the PT signals on the DME list involved in six SOCs. The two most common DMEs were pancreatitis (n = 19, ROR: 18.22) and erythema multiforme (n = 19, ROR: 3.09), which are gastrointestinal disorders and skin and subcutaneous tissue disorders, respectively. The strongest PT signal was hemolytic anemia (ROR: 47.76). The most frequent corresponding SOCs were blood and lymphatic system disorders, including febrile neutropenia (n = 16), immune thrombocytopenia (n = 8) and hemolytic anemia (n = 6).

## Discussion

To the best of our knowledge, this study represents the first analysis of ADRs associated with the combination of atezolizumab and bevacizumab in HCC patients from the FAERS database. Our findings indicate that the predominant AEs identified in the FAERS database include ascites, pyrexia, interstitial lung disease, abnormal hepatic function, adrenal insufficiency, and others, categorized under various SOCs. Patients with abnormal hepatic function and hepatic failure exhibited early failure-type profiles, which indicated that their incidence decreased over time. Ten PT signals were identified across six SOCs on the DME list. This research help to enhance the early detection and prevention of ADRs related to atezolizumab-bevacizumab combination therapy, thereby contributing to patient safety.

Most of the PTs identified in our study align with findings from previous studies and drug labels for atezolizumab and bevacizumab (Finn et al., 2020; Fukushima et al., 2023), such as interstitial lung disease, liver disorder, abnormal hepatic function, proteinuria, ascites, and hepatic failure. In the IMbrave150 trial, 22 adverse events occurred with an incidence of more than 10% among patients receiving atezolizumab and bevacizumab (Finn et al., 2020). In contrast, no PTs with an incidence exceeding 10% were identified in our study. This discrepancy is likely due to the nature of FAERS as a spontaneous reporting database, which may introduce quantitative biases stemming from diverse reporting practices. Notably, it is challenging to definitively classify disease progression as an ADR. This phenomenon may partly be attributed to hyperprogression (HP) or hyperprogressive disease (HPD). A subset of cancer patients treated with ICIs appears to experience more aggressive tumor progression, characterized by accelerated tumor proliferation and growth, leading to a shorter OS, which is regarded as HPD (Leake, 2023; Chan, 2021; Wong et al., 2019; Díaz López et al., 2024). The incidence of HPD in HCC patients is approximately 14% (Kim et al., 2021). To differentiate HPD from natural disease progression, key predictors of HPD have been identified, including higher tumor burden, elevated lactate dehydrogenase (LDH), increased neutrophil-to-lymphocyte ratio (NLR) and lower albumin levels in blood (Chan, 2021). However, predictive factors for HPD in HCC remain poorly defined, and early detection of HPD remains challenging until radiological progression or clinical deterioration occurs.

The median TTO of AEs was 43 days, with 41.17% of patients experiencing related AEs within 30 days. These findings showed that nearly half of the AEs occurred early in the treatment course, particularly those involving abnormal hepatic function and hepatic failure. Moreover, the WSP test showed that abnormal hepatic function and hepatic failure had early failure-type profiles, which suggested that the incidence of AEs decreased over time Therefore, it is imperative for healthcare providers to conduct a thorough pre-treatment evaluation of liver function in patients diagnosed with HCC. Such an assessment facilitates the identification of patients at higher risk for hepatic complications, thereby enabling the development of more personalized and cautious treatment strategies. Additionally, close monitoring of patients during early combination therapy is essential. This approach allows healthcare professionals to promptly detect and manage any emerging hepatic-related AEs, thereby mitigating the overall risk and potential severity of such events.

There are some limitations to be considered. First, because the FAERS is a spontaneous reporting system, some quantitative bias may exist due to incomplete reports or underreported cases. For example, more severe ADRs, such as life-threatening conditions or those requiring hospitalization, are generally reported more frequently than mild or moderate ones. Consequently, the FAERS database may disproportionately represent severe ADRs, while underreporting milder reactions, potentially leading to a skewed perception of the overall safety profile of a drug. Second, detailed clinical information such as OS and PFS data, therapy time and dose adjustments are missing. Third, the majority of reports originate from Asian populations, with 63.9% specifically from Japan, while there is limited data from European and African populations. Finally, the ROR merely indicates the correlation intensity of the risk of reported AEs without establishing a causal relationship between drugs and AEs. However, additional cohort studies and long-term data are essential to verify these findings. Despite the aforementioned intrinsic limitations, our study provides valuable insights into the safety profile of the combination of atezolizumab and bevacizumab, serving as a reference for future research.

## Conclusion

Overall, our study is the first to analyze the AEs associated with the combination of atezolizumab and bevacizumab in HCC patients from the FAERS database. We observed that these AEs affected multiple organ systems. Notably, patients with abnormal hepatic function and hepatic failure exhibited early failure-type profiles. Additionally, nearly half of the AEs occurred within 1 month. Our findings provide valuable pharmacological insights for clinical practice and have significant implications for early clinical intervention in managing AEs related to atezolizumab and bevacizumab combination therapy.

## Data Availability

The original contributions presented in the study are included in the article/Supplementary Material, further inquiries can be directed to the corresponding author.
