# Supplementary material for: Adverse drug reactions to atezolizumab in combination with bevacizumab in hepatocellular carcinoma patients: an analysis of the food and drug administration adverse event reporting system database
Source: Front Pharmacol. 2025 Feb 21;16:1448095. doi: 10.3389/fphar.2025.1448095 (PMC11885501; doi:10.3389/fphar.2025.1448095)
Supplement: Supplementary file 2 [file Table1.docx]

**Table S1.** Summary of equations and criteria of three algorithms for signal detection

| **Algorithms** | **Equation** | **Criteria** |
| --- | --- | --- |
| PRR | PRR=(a/(a + c))/(b/(b + d)) χ^2^=Σ((O−E)2/E); (O=a, E=(a+b)(a+c)/(a+b+c+d) | PRR≥2, χ^2^≥4, N≥3 |
| ROR | ROR=(a/b)/(c/d) | 95% CI>1, N≥2 |
| BCPNN | IC=log_2_a(a+b+c+d)/((a+c)(a+b)) | IC025>0 |

Note: ROR, reporting odds ratio: PRR, proportional reporting ratio: Cl: confidence interval; χ^2^, chi-squared; N, number of co-occurrences
